# The Impact of Age and Gender on Anterior Cruciate Ligament Injuries and Associated Knee Lesions: A Retrospective Study

**DOI:** 10.7759/cureus.68200

**Published:** 2024-08-30

**Authors:** Majed Gorayan Alrowaili

**Affiliations:** 1 Orthopedics, Faculty of Medicine, Northern Border University, Arar, SAU

**Keywords:** magnetic resonance imaging (mri), bone marrow edema (bme), collateral ligament, meniscus, anterior cruciate ligament (acl) injury

## Abstract

Objectives: This study aimed to evaluate the anterior cruciate ligament (ACL)-associated lesions in knee sports injuries in magnetic resonance imaging (MRI) and the effect of genders and ages on the patterns of the associated lesions in Arar, Northern Border region, Saudi Arabia.

Methods: This retrospective cohort study enrolled MRI of knee sports injuries with diagnosed ACL lesions during the period from January 2018 to December 2023 in Prince Abdulaziz Bin Musaed Hospital and Alkhibrah Health Center in Arar.

Results: A total of 505 knee MRI images were enrolled in the study. There were 104 (20.5%) females and 401 (79.5%) males with an average age of 34.5 years (range: 10-85 years) in this study. ACL lesions were reported in 191 (37.8%) cases. ACL was reported to be associated with other knee lesions in 185 (96.8%) cases. Joint effusion and posterior horn medial meniscus (PHMM) lesions were the most associated lesions found in 112 (58.9%) and 108 (56.5%) cases, respectively. Aging was found to significantly increase the incidence of PHMM and joint effusion associated with ACL tears, with estimated relative risks of 1.4 and 1.5 (odds ratio: 2.19 and 2.6), respectively. Also, the female gender was found to significantly increase the incidence of PHMM and associated ligament injuries with estimated relative risks of 1.5 and 4.1 (odds ratio: 3.6 and 5.1), respectively.

Conclusion: Tears of ACL are prevalent patterns of knee sports injuries with different types of associated injuries, which can be affected by the ages and genders of the patients.

## Introduction

The anterior cruciate ligament is the most important knee-stabilizing ligament, which helps to sustain stability and coordination of the knee joint [1]. Athletes face a heightened risk of injuries due to the rotational nature of their movements, deceleration while turning, and repetitive jumping and landing. The knee, a commonly injured site [2], comprises the following two functional joints: the tibiofemoral and patellofemoral joints [3]. The femoral condyles connect with the tibial plateau, and the medial and lateral menisci lie between them. Knee stability relies on the interplay of two menisci, muscles, ligaments, and capsules [4].

The anterior cruciate ligament (ACL) is crucial for knee stability and is frequently injured in athletes [5]. Serving as a major stabilizer against anterior tibial dislocation and a lesser stabilizer to lateral deviation during rotation movement [6], the ACL is prone to injuries, especially in young athletes [7]. Annually in the United States, about two-thirds of newly diagnosed cases undergo surgery [8,9].

ACL injuries are immediately disabling, requiring substantial rehabilitation time [5]. Associated articular injuries are common, increasing the potential for osteoarthritis to develop at an early stage after experiencing trauma, irrespective of the treatment administered [10]. While ACL repair restores rotational knee stability, its impact on preventing knee joint degeneration remains uncertain [11]. Various risk factors, including female sex, knee bony geometry, familial predisposition, and prior ACL repair, contribute to an increased risk of ACL injury [12]. However, no studies have investigated the association between ACL tears and other related injuries with respect to age and gender using magnetic resonance imaging (MRI). Therefore, the current study was conducted to evaluate the ACL-associated lesions among cases with knee MRI in the Arar Northern Border region, Saudi Arabia, and to assess the effects of sex and age on the patterns of associated lesions in MRI-diagnosed ACL lesion cases.

## Materials and methods

The current study was conducted as a retrospective cohort study after approval of its design by the local Bioethics Committee of Northern Border University (#94-23-H). The research enrolled MRI of individuals who experienced anterior cruciate ligament (ACL) tear after sports trauma in the Northern Border region, Saudi Arabia, during the period from January 2018 to December 2023 in Prince Abdulaziz Bin Musaed Hospital and Alkhibrah Health Center in Arar. MRI files and the related patient files were examined to collect the data related to the age and gender of cases of ACL lesions and all types of reported associated lesions related to the other knee structures. The collected data was anonymized and confidentiality was maintained. The collected data were anonymized and confidentiality was maintained. All Patients who had anterior cruciate ligament (ACL) injuries with MRI data were enrolled in the study. Cases with a history of knee surgery, congenital anomalies, or degenerative disorders were excluded.

MRI was performed using a 1.5 T system (Gyroscan Intera; Best, The Netherlands: Philips). The details of MRI examination of the enrolled cases are shown as supplementary data (appendix). For the classification of ACL tears, tears involving all fibers of the ACL are considered complete, while tears not involving the whole fibers of the ligament are considered partial. Menisci tears were classified following Dillon et al. [13]. Grade I was considered with intrasubstance tear signals, which are not reaching the articular surface. Grade II tears included intrasubstance tears with increased signal patterns without reaching the surface. Grade III tears are identified by abnormal signals reaching the superior or inferior articular surfaces. Regarding joint effusion (JE), a knee effusion was considered in cases of homogeneous well-defined soft tissue density in the suprapatellar recess, which can be clearly seen in the lateral radiograph [14].

Statistics

The data obtained was analyzed using the SPSS software program version 20 (Chicago, IL: IBM Corp.) or descriptive analysis and Fisher's exact test. Relative risk and odds ratio were estimated as parts of Fisher's exact test data. P-value <0.05 was considered statistically significant.

## Results

A total of 505 knee MRI images were revised for analysis of their findings. There were 104 (20.5%) females and 401 (79.5%) males with an average age of 34.5 years (range: 10-85 years). ACL lesions were reported in 191 (37.8%), with no significant gender predominance observed (34 females and 157 males; p-value: 0.11). Demographic data are shown in Table 1.

**Table 1:** Demographic data of cases enrolled in the study.

Group		Numbers	%
All cases with sport knee injuries (n=505)			
Gender	Male	401	79.5
	Female	104	20.5
Age	≤30 years	255	50.5
	>30 years	250	49.5
Cases with anterior crucial ligament tear (n=191)			
Gender	Male	157	82.2
	Female	34	17.8
Age	≤30 years	106	54.5
	>30 years	85	44.5

ACL complete tears were reported in 150 (78.5%) cases, while partial tears were reported in 25 (13%) and 16 (8.4%) cases. ACL was reported to be associated with other knee lesions in 185 (96.8%) cases, while ACL lesions were reported alone in the remaining six (3.2%) cases. Joint effusion was found in 112 (58.9%) cases. Posterior horn medial meniscus (PHMM) lesions were the most commonly associated lesions found in 108 (56.5%) cases with different grades. PHLM lesions were the most commonly associated lesions found in 13 (6.8%) cases with different grades. Collateral ligaments (medial collateral ligament {MCL} and lateral collateral ligament {LCL}) were found in 15 (7.8%) cases. Lesion of PCL was reported in three (1.5%) cases of ACL lesions. The distribution of lesions associated with ACL is shown in Table 2.

**Table 2:** Distribution of lesions associated with anterior cruciate ligament lesions among the studied cases. BME: bone marrow edema; JE: joint effusion; LCL: lateral collateral ligament; MCL: medial collateral ligament; PCL: posterior cruciate ligament; PHLM: posterior horn of the lateral meniscus; PHMM: posterior horn of the medial meniscus

Lesions	Ages				Gender				Total (n=191)	
	≤30 years, (n=106)		>31 years, (n=85)		Female (n=34)		Male (n=157)			
	n (years)	%	n (years)	%	n (years)	%	n (years)	%	n (years)	%
PHMM tear-I	24	22.6	30	35.3	12	35.3	42	24.0	54	28.3
PHMM tear-II	11	10.4	12	14.1	7	20.6	16	9.1	23	12.0
PHMM tear-III	16	15.1	15	17.6	8	23.5	23	13.1	31	16.2
PHLM tear-I	8	7.5	2	2.4	9	26.5	1	0.6	10	5.2
PHLM tear-II	2	1.9	1	1.2	2	5.9	1	0.6	3	1.6
JE mild	58	54.7	32	37.6	16	47.1	84	53.5	90	58
BME	8	7.5	10	11.7	3	8.8	15	9.5	18	9.4
JE moderate	16	15.1	8	9.4	3	8.8	19	12.1	22	16
LCL	2	1.9	5	5.9	4	11.8	3	1.7	7	3.7
MCL	3	2.8	4	4.7	2	5.9	5	2.9	7	3.7
PCL	1	0.9	2	2.4	2	5.9	1	0.6	3	1.6

The effect of patients' ages on the types of lesions associated with ACL injuries was evaluated. Aging was found to significantly (p-value=0.012) increase the incidence of PHMM association with ACL tears with an estimated relative risk of 1.4 (95% confidence interval {CI}: 1-1.8) and estimated odds ratio of 2.19 (95% CI: 1.2-3.9). Also, joint effusion was significantly more prevalent findings among cases above 30 years with an estimated relative risk of 1.5 (95% CI: 1.1-1.9) in the cases aged above 30 years with an estimated odds ratio of 2.6 (95% CI: 1.4-4.7) (Table 3).

**Table 3:** The effect of patients' ages on the types of lesions associated with anterior cruciate ligament lesions among the studied cases. *P-value <0.05 is considered significant. **P-value <0.01 is considered significant. BME: bone marrow edema; JE: joint effusion; PHLM: posterior horn of the lateral meniscus; PHMM: posterior horn of the medial meniscus

Lesions	Ages				Fisher’s exact p-value	Total (n=191)	
	≤30 years (n=106)		>31 years (n=85)				
	n	%	n	%		n	%
With PHMM	51	48.1	57	67.1	0.012*	108	56.5
Without PHMM	55	51.9	28	32.9		83	43.5
With PHLM	8	7.5	9	10.6	0.15	17	8.9
Without PHLM	98	92.5	76	89.4		174	91.1
With JE	74	69.8	40	47.1	0.0018**	114	59.7
Without JE	32	30.2	45	52.9		77	40.3
With BME	8	7.5	10	11.7	0.33	18	9.4
Without BME	98	92.5	75	88.3		173	90.6
With other ligaments tear	10	9.4	3	3.5	0.15	13	6.8
Without other ligaments tear	96	90.6	82	96.5		178	93.2

Finally, the effect of patients' gender on the types of lesions associated with ACL lesions was evaluated. Gender was found to significantly increase the incidence of PHMM association with ACL tears with an estimated relative risk of 1.5 (95% CI: 1.2-1.9) in the females’ cases with an odds ratio of 3.6 (95% CI: 1.5-8.8). Gender was also found to significantly increase the incidence of posterior horn lateral meniscus (PHLM) association with ACL tears with an estimated relative risk of 25.4 (95% CI: 5.8-43.9) in the females’ cases with an odds ratio of 37.07 (95% CI: 7.7-53.9). Additionally, associated ligament tears, such as LCL, MCL, and PCL were found to be significantly higher among females enrolled in the study with an estimated relative risk of 4.1 (95% CI: 1.7-9.9; odds ratio=5.1; 95% CI: 1.8-14.3) (Table 4).

**Table 4:** The effect of patients' genders on the types of lesions associated with anterior cruciate ligament tears in the studied cases. *P-value <0.01 is considered significant. **P-value <0.001 is considered significant. BME: bone marrow edema; JE: joint effusion; PHLM: posterior horn of the lateral meniscus; PHMM: posterior horn of the medial meniscus

Lesions	Gender				Fisher’s exact p-value	Total (n=191)	
	Females (n=34)		Males (n=157)				
	n	%	n	%		n	%
With PHMM	27	79.4	81	51.6	0.0038*	108	56.5
Without PHMM	7	20.6	76	48.4		83	43.5
With PHLM	11	32.4	2	1.3	<0.0001**	13	6.8
Without PHLM	23	67.6	155	98.7		178	93.2
With JE	19	55.9	103	65.6	0.32	122	63.9
Without JE	15	44.1	54	34.4		69	36.1
With BME	10	29.4	8	5.1	1	18	9.4
Without BME	24	70.6	149	94.9		173	90.6
With other ligaments tear	8	23.5	9	5.7	0.0034*	17	8.9
Without other ligaments tear	26	76.5	148	94.3		174	91.1

## Discussion

This study was conducted to evaluate the ACL-associated lesions among cases with knee MRI in the Arar Northern Border region, Saudi Arabia, in addition to the evaluation of sex and age effects on the patterns of the associated lesions with MRI-diagnosed ACL lesions cases. When diagnosing ACL injuries, magnetic resonance imaging (MRI) offers a high sensitivity of 92-100% and specificity of 85-100% [15]. Data revealed that ACL lesions were reported in 191 (37.8%) cases. ACL complete tears were reported in 150 (78.5%) cases, while partial tears and sprains were reported in 25 (13%) and 16 (8.4%) cases. ACL was reported to be associated with other knee lesions in 185 (96.8%) cases, while ACL lesions were reported alone in the remaining 57 (29.8%) cases. Joint effusion was found in 112 (58.9%) cases which is expected as a result of trauma of the knee joint structures [14]. PHMM lesions were the most commonly associated knee structure lesions found in 108 (56.5%) cases with different grades. PHLM lesions were found in 13 (6.8%) cases. Collateral ligaments (MCL and LCL) were found in 15 (7.8%) cases. Lesion of PCL was reported in three (1.5%) cases of ACL lesions.

The current data showed no gender-related predominance of ACL among cases of sports knee injuries. That is in accordance with the findings of the study of Quisquater et al. [16]. While other previously published data revealed that females had a two to six times higher risk for ACL tears [17,18]. This higher risk was explained by hormonal differences, which is still a matter of debate. This contradiction of data can be explained by different samples enrolled in the different studies or the different variables that affect ACL tear occurrence which can affect the outcomes of each other’s.

The current data revealed that meniscus lesions are a common finding with ACL sports injuries with more predominance of PHMM. This is in accordance with the findings of the study by Venkataraman et al. [19]. In contrast to the medial meniscus, which is less mobile because it is attached to the tibia, the lateral meniscus is more mobile. This makes the medial meniscus more vulnerable to damage when the knee joint twists [20]. The posterior horn of the medial meniscus was found to be the main injured site of the medial meniscus. This can be explained by its function as a wedge between the tibia and femur with more susceptibility to high stress with tibial translational movement [20].

Previous literature has investigated the factors affecting the pattern of the associated knee sports injuries such as ACL and the different patterns of the associated lesions of menisci and other ligament tears. The forces that sports activities performed at the time of injury may have applied to the knee can be reflected in damage patterns. It was noted that the kind of sport played had an impact on the injuries sustained. While meniscus and cartilage injuries are less common in sports like soccer and skiing, isolated ACL tears and other ligament injuries are more common. The distal leg and ski function as a long lever arm in a slip-catch mechanism, which is most likely the cause of this damage pattern [21]. Compared to soccer, American football has a higher risk of multiligament injuries. The energy and biomechanics at the scene of the injury might be the cause. Another possible explanation could be the larger ratio of contact to noncontact ACL injuries in American football as opposed to soccer [22]. Compared to soccer, basketball has a higher risk of lateral meniscus tears and cartilage damage. According to Krosshaug et al., this could be the outcome of the pressures delivered to the knee at the moment of contact in most cases involving landing after jumping [23]. Because team handball and basketball involve more cut and plant motions than soccer, the elevated risks for lateral meniscus injuries are similar for both sports. This is also consistent with a large cohort study that indicated the only male athletes at higher risk of lateral meniscus injury were male team handball players [24]. This could be explained by the fact that male team handball has more elite and intermediate athletes than other sports [24].

Furthermore, the time to surgery was considered a limiting factor for ACL-associated lesions, as research has shown that the probability of meniscus and cartilage damage increases with the duration between the injury and surgery [25,26]. It was discovered, although, that it did not significantly alter the association between the majority of the concurrent injuries examined and the sports practiced at the time of injury. After accounting for the time to surgery (less than 20%, which is taken as the threshold of a large confounder), the majority of the estimations changed very little.

It was assessed how a patient's age affected the kinds of lesions connected to ACL lesions. In the recruited cases with MRI, it was discovered that aging considerably increased the incidence of PHMM connection with ACL tears, with an estimated relative risk of 1.4 in cases older than 30 years (odds ratio=2.19). This is in accordance with Astur et al. who reported a higher incidence of sports injuries and meniscal lesions above the age of 30 years [27]. Furthermore, joint effusion was shown to be much more common in individuals over 30 years of age, with an estimated relative risk of 1.5 (odds ratio=2.6) in these cases. This higher incidence of knee effusion among females was in accordance with the findings of the study by Hung et al., which was mainly attributed to the expected higher body mass index among enrolled female cases [28].

Lastly, the relationship between a patient's gender and the types of lesions associated with ACL injuries was assessed. In the recruited cases with MRI, gender was observed to significantly increase the incidence of PHMM with ACL tears, with an estimated relative risk of 1.5 in the cases of females (odds ratio=3.6). In the MRI cases that were recruited, gender was also observed to significantly increase the incidence of PHLM connection with ACL tears, with an estimated relative risk of 25.4 in the instances of females (odds ratio=37.07). Furthermore, it was discovered that female participants in the study had considerably greater rates of related ligament tears, such as LCL, MCL, and PCL, with an estimated relative risk of 4.1 (odds ratio=5.1). These higher risks can be explained due to different factors including hormonal background, body mass index, smaller size of ligaments among females with more expected stress on the ligaments with sports injuries, and the difference in shape and width of the intercondylar notch among both genders [29].

The main limitation of the current study is that we don't have any footage of the injury episodes, thus it's only theoretical to speculate on the precise biomechanical injury mechanism. The registries did not note if the injuries were noncontact or contact. Furthermore, as the study's design is cross-sectional, it is unable to discuss how the concurrent injuries and activities under investigation relate to one another in time. Also, the registries did not include data about the participants' body mass indices which can add further data analysis. Additionally, the data were based on a local two-center study with a higher enrollment of male cases, which may be attributed to cultural factors and less participation in sports among females. However, it is plausible that the injuries either preceded the ACL tear or occurred later and were only identified at the time of the ACLR. On the other hand, we believe that the inability to pinpoint the exact moment of injury is not unique to any of the activities under investigation.

This study's main strength is the vast number of patients it included and the reliable results it produced. Therefore, it seems reasonable to think that the MRI injury patterns could be a reflection of the stresses that participating in sports activities imparted to the knee. Other strengths of this study include the data sources that were employed, the comparable formats and methods of data collection, and the previously noted overlaps in the definitions that were used.

## Conclusions

The current study concluded that ACL lesions are a prevalent pattern of knee sports injuries in the Northern Border region, Saudi Arabia, without gender predominance. ACL injuries were commonly associated with other knee lesions. Joint effusion was the most common co-finding, followed by PHMM lesions of different grades. PHLM lesions and collateral ligaments (MCL and LCL) were found in less than 10% of ACL lesion cases. Aging was found to significantly increase the incidence of PHMM and joint effusion associated with ACL injuries. Additionally, the female gender was found to significantly increase the incidence of PHMM and associated ligament injuries. These findings highlight the importance of considering ACL lesions and co-founding lesions in the choice of treatment plans for sports injuries. Moreover, associated lesions are expected to affect management outcomes. The data also support the importance of increasing awareness about ACL lesions among athletes.

## Appendices

The details of the MRI examination of the enrolled cases

MRI was performed using a 1.5 T system (Gyroscan Intera; Best, The Netherlands: Philips) with a circular polarized surface coil using identical sequences for all participants and all time points. The MRI pulse sequence protocol included a sagittal 3D water excitation Fast Low Angle Shot (FLASH) sequence with a repetition time (TR)/echo time (TE)/flip angle of 20 ms/7.9 ms/25° and a sagittal T2-weighted 3D gradient echo (GRE) sequence with TR/TE/flip angle of 20 ms/15 ms/50°. Both series were acquired with a 15 x 15 cm field of view (FOV), 1.5 mm slice thickness, and 0.29 x 0.29 mm pixel size. In addition, a sagittal and coronal dual echo turbo spin echo (DETSE) sequence, with a TR/TE of 2900/15 ms and 80 ms, 15 x 15 cm FOV, 3 mm slice thickness, 0.6 mm gap and 0.59 x 0.59 mm pixel size, and sagittal and coronal short tau inversion recovery (STIR) sequences with a TR/TE/TI of 2900 ms/15 ms/160 ms, 15 x 15 cm FOV, 3 mm slice thickness, 0.6 mm gap and 0.29 x 0.29 mm pixel size were acquired.
